# Knowledge, Attitudes, Practices, and Illness Perception of Female Outpatients With Chronic Pelvic Pain

**DOI:** 10.1155/prm/5017608

**Published:** 2026-10-05

**Authors:** Yiran Qiu, Yiming Wang, Fan Li, Guangting Mao, Chubin Xing, Xiaoyu Wang, Jinpeng Qiu

**Affiliations:** ^1^ Department of Hand and Foot Surgery Orthopaedics Center The First Hospital of Jilin University Changchun 130021 China jlu.edu.cn; ^2^ Department of Anesthesiology The First Hospital of Jilin University Changchun 130012 China jlu.edu.cn; ^3^ Department of Anesthesiology Lequn Branch The First Hospital of Jilin University Changchun Jilin 130031 China jlu.edu.cn; ^4^ Department of Obstetrics The First Hospital of Jilin University Changchun 130021 China jlu.edu.cn

**Keywords:** attitude, chronic pelvic pain, female outpatients, knowledge, practice

## Abstract

**Purpose:**

Chronic pelvic pain (CPP), defined as pelvic discomfort lasting ≥ 6 months, substantially impairs women’s quality of life. This study evaluated CPP‐related knowledge, attitudes, practices (KAP), and illness perception among female outpatients.

**Methods:**

A total of 597 female outpatients from the First Hospital of Jilin University were included between September and October 2024. Data were collected using a self‐designed KAP questionnaire, the Brief Illness Perception Questionnaire (B‐IPQ), and the Physical Activity Rating Scale‐3. Latent profile analysis (LPA) was used to identify illness perception profiles, and path analysis using observed total scores was employed to analyze the associations between KAP variables.

**Results:**

Analysis of 597 responses revealed key characteristics: 31.2% were aged 45–54, 38.7% had pain for 2–5 years, and 80.2% reported low physical activity. Mean KAP scores were indicative of inadequate knowledge, positive attitudes, and moderate practices. LPA identified three illness perception classes. The high illness perception group had the highest B‐IPQ and significantly higher proportions of severe pain (≥ 5), prior pelvic surgery, and low physical activity. Path analysis showed significant direct associations: Knowledge was positively associated with Attitude (*β* = 0.394) and Practice (*β* = 0.378), and Attitude was associated with Practice (*β* = 0.148). Knowledge also showed a significant indirect association with Practice through Attitude (*β* = 0.058), while the total association of Knowledge with Practice was *β* = 0.436. All paths were statistically significant (*p* < 0.001). Multivariate logistic regression further identified knowledge, attitude, medium illness perception (Class 3), and experience of pelvic floor muscle exercises as independent predictors of good practice (practice score ≥ 60%).

**Conclusion:**

Overall, participants demonstrated inadequate knowledge, positive attitudes, and moderate practices. Participants with high illness perception demonstrated the highest KAP scores despite more severe symptoms. These findings underscore the need for targeted educational interventions to enhance CPP‐related KAP in this population.

## 1. Introduction

CPP is a multifactorial condition with various etiologies, including gynecological, gastrointestinal, urological, musculoskeletal, and psychological causes [1]. Epidemiological estimates indicate that CPP affects approximately 26% of the global female population and 4% of women of reproductive age in developed countries [1, 2]. In China, a hospital‐based community study reported that 15.3% of women experience CPP [3]. The quality of life for affected women is often severely compromised, as CPP interferes with routine activities, social relationships, and work productivity [4]. Physical function and mental health are frequently impaired, with women reporting limitations in mobility and emotional stress [5, 6].

Given the significant health burden of CPP, which is compounded by its incompletely understood mechanisms and the persistent challenges in clinical treatment [1], it is crucial to incorporate both medical and psychosocial support tailored to the specific needs of the affected women. Self‐management strategies, such as pelvic floor exercises, medical counseling, and dietary modifications, can provide some relief and complement clinical treatments [4]. The knowledge, attitude, and practice (KAP) theoretical framework provides a systematic approach to understanding these health behaviors [7]. First proposed in the late 1950s and early 1960s, the KAP model posits that behavioral change proceeds through three sequential stages: acquiring knowledge, forming attitudes, and adopting practices [8]. Thus, understanding the KAP of female outpatients regarding CPP is essential for designing patient‐centered interventions.

Furthermore, patients’ illness perceptions, particularly beliefs regarding treatment efficacy, personal control, and illness coherence, play a pivotal role in influencing adherence to CPP management [9]. Those who perceive greater control over their symptoms and have a coherent understanding of their condition are more likely to follow treatment regimens, engage in recommended lifestyle modifications, and maintain long‐term self‐management behaviors. Moreover, illness perceptions shape emotional responses, which may either motivate or discourage active participation in care [10]. Beyond their role in shaping health behaviors, illness perceptions have also been linked to measurable alterations in pain processing [11]. In women with chronic pelvic pain (CPP) syndromes, negative illness perceptions have been shown to be associated with a pronociceptive pain modulation profile characterized by enhanced temporal summation and less efficient conditioned pain modulation, as well as augmented clinical pain [12]. In addition, myofascial physical therapy has been reported to produce multisystemic biopsychophysiological effects in this population, highlighting the interconnection between perceptual, neurophysiological, and behavioral dimensions of CPP [13]. Accordingly, exploring the interplay between illness perception and patients’ KAP is vital to identify distinct subgroups that may benefit from tailored interventions aimed at optimizing both medical and behavioral outcomes.

To date, no KAP studies have addressed CPP among female outpatients in China, with a focus on their illness perceptions. Existing studies have focused on KAP related to pelvic floor dysfunction, a significant contributor to CPP. For instance, Chinese obstetrical healthcare providers demonstrated substantial knowledge and positive attitudes toward pelvic floor dysfunction but rarely provided related guidance to patients [14]. To address these gaps, the present study aimed to investigate the KAP and illness perceptions of female outpatients with CPP. The study hypotheses were as follows: (1) female outpatients’ knowledge is positively associated with their attitudes toward CPP, (2) knowledge is positively associated with their self‐management practices, and (3) attitudes are positively associated with their practices. Our findings can offer insights into developing targeted educational and behavioral interventions that support better outcomes for women with CPP. Moreover, by identifying modifiable factors associated with good self‐management practice, this study provides actionable evidence for clinicians to prioritize specific intervention targets in routine care.

## 2. Materials and Methods

### 2.1. Study Design and Participants

This cross‐sectional survey was conducted between September and October 2024 at The First Hospital of Jilin University. Female outpatients diagnosed with CPP were recruited from the gynecology and anesthesiology (pain management) outpatient departments for the study. The inclusion criteria were: (1) women aged 30–65 years and (2) those experiencing pelvic pain persisting for over 6 months. The exclusion criteria were: (1) individuals with purely cyclical menstrual pain (i.e., pain occurring exclusively during menstruation without intermenstrual symptoms), as the study focused on noncyclical or persistent pelvic pain; (2) pain attributable to tumors; (3) lack of informed consent; and (4) questionnaires containing logical inconsistencies, incomplete responses, or errors.

The study protocol was approved by the Ethics Committee of The First Hospital of Jilin University (approval number: 24K220‐001), and informed consent was obtained from all participants.

### 2.2. Questionnaire

The questionnaire (supporting information) was developed in accordance with the Expert Consensus on Clinical Management of CPP in Women [15] and prior research [16]. Initial items were generated by the study team based on these sources and clinical experience. Three senior specialists then reviewed all items for preliminary content validity, assessing correctness, relevance, and clarity. A pilot test involving 27 participants was subsequently conducted, yielding Cronbach’s α coefficients of 0.926, 0.481, and 0.882 for the KAP dimensions, respectively. The relatively low reliability of the attitude dimension in the pilot phase was attributable to the small number of scored items (four items) and the heterogeneity of attitudinal constructs assessed [17]. Based on pilot feedback, minor wording revisions were made to improve readability for the patient population (face validity), and one item regarding experience of pelvic organ surgery was added to the demographic section. No items were removed. In the formal survey (*n* = 597), the overall Cronbach’s α was 0.869, with values of 0.879, 0.726, and 0.744 for the KAP dimensions, respectively. The finalized Chinese‐language questionnaire comprised six sections: demographic information, knowledge dimension, attitude dimension, practice dimension, physical activity level scale, and the Brief Illness Perception Questionnaire (B‐IPQ).

The knowledge dimension included 6 questions with 12 items, each scored as follows: “Aware” (2 points), “Partially aware” (1 point), and “Unaware” (0 points). The total score for the knowledge dimension ranged from 0 to 24. For the knowledge dimension, Cronbach’s α was reported as a reference indicator of internal consistency; however, given that knowledge items assess factual awareness across diverse content areas rather than a single underlying trait, internal consistency may not fully capture the measurement quality of this domain [18]. The attitude dimension consisted of five self‐designed items for CPP, of which four (A1–A3 and A5) were scored; item A4 was descriptive only, as it did not indicate a positive or negative attitude. The four scored items were rated on a five‐point Likert scale ranging from “Strongly agree” (5 points) to “Strongly disagree” (1 point). The total score for the attitude dimension ranged from 4 to 20. The practice dimension included six items; items 1–5 were scored on a five‐point Likert scale from “Always” (5 points) to “Never” (1 point), while item 6 was descriptive only. The total score for the practice dimension ranged from 5 to 25. Scoring was based on Bloom’s cutoff criteria, with scores of 80%–100% categorized as good, 60%–79% as moderate, and below 60% as poor [19].

The Physical Activity Rating Scale‐3 is a structured questionnaire utilized to assess the frequency, intensity, and duration of physical activities in daily life. Physical activity levels were categorized as low, moderate, or high [20]. The B‐IPQ is a validated tool designed to assess patients′ cognitive and emotional representations of illness as well as their perceived understanding [21]. It comprises eight core items structured across three dimensions: cognitive representation (items 1–5), emotional representation (items 6 and 8), and illness comprehension (item 7). The cognitive dimension evaluates beliefs regarding the illness’s consequences, expected duration (timeline), personal and treatment control, and symptom identity. The emotional dimension captures the patient’s level of concern and emotional distress associated with the illness. The comprehension dimension assesses the extent to which individuals believe they understand their condition. Each item is rated on a 0–10 scale, with higher scores typically reflecting greater perceived illness threat, except for positively framed items such as personal control, treatment control, and understanding, which are reverse scored when calculating the B‐IPQ total score. Notably, for the latent profile analysis (LPA) (described below), raw (nonreversed) item scores were used, such that higher scores on items 3 (personal control), 4 (treatment control), and 7 (understanding) indicated greater perceived control or comprehension.

Female outpatients with CPP were recruited through convenience sampling. Patients provided written informed consent prior to participation. Research staff clarified questionnaire items when necessary, ensuring that their explanations did not influence participants’ responses. Upon completion, the research team reviewed the questionnaires for completeness, internal consistency, and logical coherence.

### 2.3. Statistical Analysis

All analyses were conducted using Stata 18.0 (Stata Corp LP, USA). Continuous data (e.g., KAP scores) were expressed as mean ± standard deviation (SD) and compared using independent samples *t*‐tests or one‐way ANOVA. Categorical variables (e.g., demographic characteristics) were represented as *n* (%). To examine patterns of illness perception, raw (nonreversed) B‐IPQ item scores were subjected to LPA. Model selection was guided by several criteria: (1) lower values of Log‐Likelihood (LogLik), Akaike Information Criterion (AIC), Bayesian Information Criterion (BIC), and Integrated Completed Likelihood (ICL); and (2) higher entropy, with values > 0.80 indicating strong class separation. In addition, average posterior class‐membership probabilities were evaluated to assess classification certainty. Models with two to five latent classes were evaluated. The optimal number of classes was determined by balancing statistical fit indices, classification quality, and clinical interpretability. Sociodemographic characteristics and KAP scores were then compared across latent classes, and item‐level KAP distributions were profiled within each B‐IPQ class. Correlations among KAP scores were assessed using Spearman’s correlation analysis. Guided by the KAP theoretical framework, path analysis using observed total scores for KAP was employed to explore whether attitude statistically mediates the association between knowledge and practice. Both direct and indirect associations were calculated. Furthermore, to identify factors associated with good practice, practice scores were dichotomized using Bloom’s cutoff criterion (≥ 60% of the total score, i.e., ≥ 15 points). This threshold was selected based on the widely adopted Bloom’s cutoff framework, which classifies scores of ≥ 80% as good, 60%–79% as moderate, and < 60% as poor [19]. To examine the robustness of the dichotomized findings, a sensitivity analysis using raw practice scores as a continuous outcome in linear regression was also performed. Variables significantly associated with good practice in univariate logistic regression (*p* < 0.05) were entered into a multivariate logistic regression model. Collinearity was assessed using tolerance and variance inflation factor (VIF), with VIF < 10 indicating acceptable collinearity levels. All statistical analyses were two‐sided, with *p* < 0.05 considered statistically significant.

## 3. Results

A total of 659 questionnaires were initially collected, of which 62 were excluded due to incomplete data (*n* = 38) or conflicting responses (*n* = 24). Consequently, 597 valid cases remained. As summarized in Table 1, 31.2% of participants were aged between 45 and 54 years. The majority were married (91.0%), resided in urban areas (67.2%), and held an associate or bachelor’s degree (53.4%). Most participants had not undergone a cesarean section (65.2%), did not perform pelvic floor muscle exercises post‐childbirth (79.4%), and reported low physical activity levels (≤ 19 points) (80.2%) (Supporting Table 1).

**TABLE 1:** Demographic characteristics of participants.

Characteristics	*N* (%)	Knowledge	*p*	Attitude	*p*	Practice	*p*
		Mean ± SD		Mean ± SD		Mean ± SD	
Total score	597 (100.0)	7.94 ± 4.52		16.19 ± 2.09		15.17 ± 3.34	
Age, years			< 0.001		0.438		< 0.001
< 35	80 (13.4)	9.91 ± 6.83		16.42 ± 2.64		14.95 ± 4.22	
35–44	183 (30.7)	8.21 ± 4.06		16.15 ± 2.05		15.93 ± 3.14	
45–54	186 (31.2)	7.81 ± 4.18		16.25 ± 2.05		15.01 ± 3.33	
≥ 55	148 (24.8)	6.72 ± 3.41		16.05 ± 1.85		14.53 ± 2.89	
BMI (kg/m^2^)			0.402		0.189		0.023
< 18.5	23 (3.9)	7.17 ± 4.59		15.52 ± 2.11		15.26 ± 3.37	
18.50–23.99	343 (57.5)	7.99 ± 3.96		16.22 ± 1.91		15.32 ± 3.20	
24.00–27.99	193 (32.3)	7.84 ± 4.98		16.12 ± 2.31		15.18 ± 3.46	
≥ 28.0	38 (6.4)	8.53 ± 6.50		16.68 ± 2.43		13.66 ± 3.68	
Marital status			0.546		0.022		0.195
Married	543 (91.0)	8.00 ± 4.52		16.27 ± 2.09		15.28 ± 3.27	
Single	24 (4.0)	6.71 ± 4.67		15.21 ± 2.40		13.50 ± 4.14	
Divorced	27 (4.5)	7.63 ± 4.08		15.44 ± 1.42		14.22 ± 3.27	
Other	3 (0.5)	11.00 ± 7.00		16.67 ± 2.52		16.00 ± 7.00	
Gravidity			0.305		< 0.001		0.012
0	24 (4.0)	6.42 ± 4.99		15.38 ± 2.89		12.83 ± 4.22	
1	270 (45.2)	7.97 ± 4.46		16.42 ± 1.98		15.48 ± 3.28	
2	224 (37.5)	7.83 ± 4.01		15.79 ± 1.98		15.27 ± 3.21	
3	48 (8.0)	7.90 ± 4.75		16.35 ± 2.29		14.46 ± 3.06	
> 3	31 (5.2)	9.84 ± 6.93		17.45 ± 2.05		14.58 ± 3.73	
Parity			0.130		0.021		0.002
0	35 (5.9)	6.26 ± 4.49		15.60 ± 2.48		13.54 ± 4.01	
1	448 (75.0)	7.91 ± 4.17		16.18 ± 1.86		15.38 ± 3.14	
2	99 (16.6)	8.55 ± 5.04		16.22 ± 2.71		15.16 ± 3.65	
3	15 (2.5)	8.93 ± 8.90		17.80 ± 2.37		12.53 ± 3.58	
Experience of cesarean section			< 0.001		0.002		0.115
Yes	208 (34.8)	9.19 ± 5.49		16.53 ± 2.34		15.52 ± 3.48	
No	389 (65.2)	7.38 ± 3.66		16.05 ± 1.86		15.12 ± 3.14	
Experience of pelvic floor muscle exercises after childbirth			< 0.001		0.174		< 0.001
Yes	123 (20.6)	9.97 ± 3.98		16.39 ± 1.75		17.26 ± 2.93	
No	474 (79.4)	7.51 ± 4.50		16.18 ± 2.14		14.71 ± 3.15	
Residence			< 0.001		< 0.001		< 0.001
Urban	401 (67.2)	8.94 ± 4.62		16.47 ± 2.09		15.72 ± 3.33	
Rural	97 (16.2)	6.07 ± 4.29		15.62 ± 2.33		14.40 ± 3.59	
Suburban	99 (16.6)	5.77 ± 2.61		15.63 ± 1.58		13.66 ± 2.45	
Average commute time to the hospital for treatment, hours			< 0.001		0.001		0.003
< 1	158 (26.5)	8.75 ± 4.90		16.55 ± 2.15		14.93 ± 3.25	
1.00–1.99	293 (49.1)	8.23 ± 4.30		16.24 ± 1.95		15.60 ± 3.42	
2.00–2.99	108 (18.1)	6.44 ± 3.96		15.52 ± 2.18		14.47 ± 3.05	
≥ 3	38 (6.4)	6.68 ± 4.97		16.26 ± 2.29		14.76 ± 3.53	
Ethnicity			0.742		0.658		0.269
Han	559 (93.6)	7.97 ± 4.57		16.19 ± 2.11		15.21 ± 3.37	
Minority ethnic group	38 (6.4)	7.50 ± 3.82		16.26 ± 1.88		14.53 ± 2.94	
Religious belief			0.178		0.054		0.238
Yes	63 (10.6)	7.60 ± 4.93		15.62 ± 2.32		14.84 ± 3.96	
No	534 (89.4)	7.99 ± 4.47		16.26 ± 2.06		15.20 ± 3.26	
Education			< 0.001		0.055		< 0.001
Primary school or below	25 (4.2)	4.72 ± 4.06		15.80 ± 2.78		13.96 ± 3.43	
Middle school/high school/technical school	195 (32.7)	6.72 ± 3.34		16.07 ± 2.06		14.10 ± 2.84	
Associate/bachelor’s degree	319 (53.4)	8.01 ± 4.14		16.15 ± 1.99		15.68 ± 3.29	
Master’s degree or above	58 (9.7)	13.07 ± 6.07		16.98 ± 2.30		16.45 ± 4.01	
Physically demanding occupations			< 0.001		0.023		< 0.001
Yes	34 (5.7)	6.41 ± 4.72		15.62 ± 2.73		13.62 ± 3.70	
No	478 (80.1)	8.46 ± 4.64		16.33 ± 2.08		15.45 ± 3.41	
Unsure	85 (14.2)	5.66 ± 2.56		15.66 ± 1.76		14.21 ± 2.32	
Monthly income, Chinese yuan			< 0.001		< 0.001		< 0.001
< 2000	31 (5.2)	5.10 ± 2.33		15.19 ± 1.74		13.00 ± 2.72	
2000–5000	228 (38.2)	6.54 ± 3.31		16.00 ± 2.09		14.16 ± 2.75	
5000–10,000	184 (30.8)	9.17 ± 4.25		16.35 ± 1.73		16.59 ± 3.20	
10,000–20,000	26 (4.4)	10.77 ± 4.61		16.31 ± 1.98		16.27 ± 3.83	
> 20,000	22 (3.7)	16.41 ± 8.57		18.09 ± 2.96		15.45 ± 5.65	
Prefer not to disclose	106 (17.8)	7.22 ± 3.59		16.20 ± 2.34		15.16 ± 3.13	
Duration of pelvic pain, years			0.241		0.117		0.357
< 2	127 (21.3)	8.03 ± 4.53		16.09 ± 2.33		15.36 ± 3.32	
2.00–4.99	231 (38.7)	8.19 ± 4.89		16.10 ± 2.02		15.24 ± 3.49	
5.00–9.99	147 (24.6)	8.01 ± 4.30		16.54 ± 1.91		14.84 ± 3.26	
≥ 10	92 (15.4)	7.11 ± 3.80		16.00 ± 2.18		15.23 ± 3.11	
Causes of pelvic pain			0.310		< 0.001		0.240
Gynecological condition	426 (71.4)	8.21 ± 4.74		16.44 ± 2.04		15.31 ± 3.33	
Gastrointestinal condition	16 (2.7)	7.75 ± 5.42		16.50 ± 2.31		15.50 ± 3.52	
Urological condition	17 (2.8)	7.00 ± 4.66		16.18 ± 1.74		15.53 ± 4.37	
Myofascial pain	7 (1.2)	8.57 ± 2.23		17.00 ± 2.38		16.14 ± 2.91	
Sociopsychological condition	6 (1.0)	8.33 ± 3.27		13.83 ± 4.83		15.00 ± 5.59	
Neurological condition	34 (5.7)	7.18 ± 2.59		15.18 ± 1.17		14.06 ± 2.33	
Others	91 (15.2)	7.11 ± 3.94		15.46 ± 2.03		14.71 ± 3.32	
Self‐rated pain			0.001		0.087		0.040
1	36 (6.0)	6.94 ± 5.05		16.31 ± 2.95		13.92 ± 3.56	
2	83 (13.9)	8.33 ± 5.38		16.52 ± 2.61		14.93 ± 3.74	
3	68 (11.4)	7.59 ± 5.26		15.54 ± 2.67		14.41 ± 3.91	
4	59 (9.9)	7.63 ± 3.81		16.24 ± 1.47		15.81 ± 3.07	
5	99 (16.6)	7.22 ± 4.43		16.23 ± 2.02		15.24 ± 3.36	
6	129 (21.6)	7.71 ± 3.83		16.06 ± 1.65		15.53 ± 3.04	
7	82 (13.7)	8.72 ± 3.51		16.28 ± 1.57		15.34 ± 2.78	
8	41 (6.9)	10.05 ± 5.18		16.56 ± 1.91		15.37 ± 3.28	
Experience of pelvic organ surgery			0.313		0.074		0.164
None	289 (48.4)	7.87 ± 4.29		16.09 ± 2.19		14.96 ± 3.53	
Gynecological/obstetric surgery	255 (42.7)	8.24 ± 4.84		16.41 ± 1.99		15.26 ± 3.06	
Urological surgery	15 (2.5)	6.93 ± 3.51		16.20 ± 1.15		16.80 ± 2.86	
Colorectal surgery	24 (4.0)	6.38 ± 3.00		16.08 ± 1.61		15.17 ± 2.87	
Have undergone surgery, details unclear	14 (2.3)	7.86 ± 5.92		14.57 ± 2.65		16.00 ± 4.98	
Physical Activity Level Scale			< 0.001		0.038		< 0.001
Low	479 (80.2)	7.38 ± 4.05		16.10 ± 2.05		14.83 ± 3.18	
Moderate	96 (16.1)	9.43 ± 4.98		16.53 ± 1.76		16.27 ± 3.35	
High	22 (3.7)	13.68 ± 6.53		16.68 ± 3.63		17.68 ± 4.65	

The duration of pelvic pain for 38.7% of participants ranged from 2 to 4.99 years, and 21.6% reported a pain severity score of 6. Regarding the perceived causes of pelvic pain, 71.4% attributed it to a gynecological condition (Supporting Table 1).

LPA with two to five latent classes were evaluated. The three‐class model was selected based on relatively low LogLik, AIC, BIC, and ICL values, and an entropy approaching 0.80 (Supporting Table 2). Although AIC, BIC, and adjusted BIC continued to decrease toward the four‐class solution, the classification‐oriented indices uniformly favored the three‐class model: ICL reached its optimum at three classes (−9815.9 vs. −9886.4 for four classes), entropy was highest at three classes (0.79 vs. 0.72), and the average posterior class‐membership probabilities were uniformly higher and more homogeneous (0.86–0.92 vs. 0.82–0.88). The improvement in BIC also leveled off markedly after three classes (Δ = 98.6 for 2 ⟶ 3 vs. 24.2 for 3 ⟶ 4), indicating diminishing returns. The four‐class solution did not yield an additional clinically interpretable profile. The three‐class model was therefore retained on the basis of its superior classification quality, parsimony, and interpretability. The model classified participants into class 1 (low illness perception, 31.49%), class 2 (high illness perception, 10.55%), and class 3 (medium illness perception, 57.96%). Because raw (nonreversed) B‐IPQ item scores were used in the LPA, higher scores on items 3 (personal control), 4 (treatment control), and 7 (understanding) reflected greater perceived control or comprehension. As shown in Supporting Figure 1, the patients in the high illness perception class had the highest scores across all B‐IPQ items, indicating not only the greatest perceived illness threat (items 1, 2, 5, 6, and 8) but also the highest confidence in personal and treatment control and the greatest illness comprehension (items 3, 4, and 7), whereas class 1 had the lowest scores across all items. The comparison of demographic characteristics among the classes is shown in Table 2. The smallest class (class 2) comprised 63 participants (10.55%). Despite its relatively small size, this class was characterized by a distinct and clinically coherent profile, with the highest B‐IPQ scores, the highest proportions of severe pain (≥ 5), prior pelvic surgery, and low physical activity, supporting its interpretability and stability.

**TABLE 2:** Characteristics of participants across classes of brief illness perception questionnaire (B‐IPQ).

	Group 1 (*N* = 188) mean ± SD/*N* (%)	Group 2 (*N* = 63) mean ± SD/*N* (%)	Group 3 (*N* = 346) mean ± SD/*N* (%)	*p*
Age, years				< 0.001
< 35	15 (7.98)	29 (46.03)	36 (10.4)	
35–44	52 (27.66)	18 (28.57)	113 (32.66)	
45–54	60 (31.91)	12 (19.05)	114 (32.95)	
≥ 55	61 (32.45)	4 (6.35)	83 (23.99)	
BMI (kg/m^2^)				0.076
< 18.5	10 (5.32)	1 (1.59)	12 (3.47)	
18.50–23.99	115 (61.17)	30 (47.62)	198 (57.23)	
24.00–27.99	57 (30.32)	24 (38.1)	112 (32.37)	
≥ 28.0	6 (3.19)	8 (12.7)	24 (6.94)	
Marital status				0.094
Married	167 (88.83)	58 (92.06)	318 (91.91)	
Single	6 (3.19)	5 (7.94)	13 (3.76)	
Divorced	13 (6.91)	0 (0)	14 (4.05)	
Other	2 (1.06)	0 (0)	1 (0.29)	
Gravidity				0.001
0	10 (5.32)	3 (4.76)	11 (3.18)	
1	88 (46.81)	24 (38.1)	158 (45.66)	
2	78 (41.49)	20 (31.75)	126 (36.42)	
3	9 (4.79)	6 (9.52)	33 (9.54)	
> 3	3 (1.6)	10 (15.87)	18 (5.2)	
Parity				< 0.001
0	10 (5.32)	6 (9.52)	19 (5.49)	
1	152 (80.85)	30 (47.62)	266 (76.88)	
2	26 (13.83)	21 (33.33)	52 (15.03)	
3	0 (0)	6 (9.52)	9 (2.6)	
Experience of cesarean section				< 0.001
Yes	46 (24.47)	36 (57.14)	126 (36.42)	
No	142 (75.53)	27 (42.86)	220 (63.58)	
Experience of pelvic floor muscle exercises after childbirth				0.003
Yes	23 (12.23)	14 (22.22)	86 (24.86)	
No	165 (87.77)	49 (77.78)	260 (75.14)	
Residence				< 0.001
Urban	106 (56.38)	46 (73.02)	249 (71.97)	
Rural	31 (16.49)	14 (22.22)	52 (15.03)	
Suburban	51 (27.13)	3 (4.76)	45 (13.01)	
Average commute time to the hospital for treatment, hours				0.134
< 1	44 (23.40)	15 (23.81)	99 (28.61)	
1.00–1.99	89 (47.34)	33 (52.38)	171 (49.42)	
2.00–2.99	45 (23.94)	8 (12.70)	55 (15.90)	
≥ 3	10 (5.32)	7 (11.11)	21 (6.07)	
Ethnicity				0.010
Han	169 (89.89)	63 (100)	327 (94.51)	
Minority ethnic group	19 (10.11)	0 (0)	19 (5.49)	
Religious belief				< 0.001
Yes	37 (19.68)	2 (3.17)	24 (6.94)	
No	151 (80.32)	61 (96.83)	322 (93.06)	
Education				< 0.001
Primary school or below	5 (2.66)	1 (1.59)	19 (5.49)	
Middle school/high school/technical school	73 (38.83)	10 (15.87)	112 (32.37)	
Associate/bachelor’s degree	104 (55.32)	39 (61.9)	176 (50.87)	
Master’s degree or above	6 (3.19)	13 (20.63)	39 (11.27)	
Physically demanding occupations				0.002
Yes	5 (2.66)	1 (1.59)	28 (8.09)	
No	151 (80.32)	60 (95.24)	267 (77.17)	
Unsure	32 (17.02)	2 (3.17)	51 (14.74)	
Monthly income, Chinese yuan				< 0.001
< 2000	22 (11.7)	0 (0)	9 (2.6)	
2000–5000	92 (48.94)	9 (14.29)	127 (36.71)	
5000–10,000	53 (28.19)	12 (19.05)	119 (34.39)	
10,000–20,000	9 (4.79)	5 (7.94)	12 (3.47)	
> 20,000	3 (1.6)	11 (17.46)	8 (2.31)	
Prefer not to disclose	9 (4.79)	26 (41.27)	71 (20.52)	
Duration of pelvic pain, years				< 0.001
< 2	38 (20.21)	24 (38.1)	65 (18.79)	
2.00–4.99	80 (42.55)	28 (44.44)	123 (35.55)	
5.00–9.99	43 (22.87)	8 (12.7)	96 (27.75)	
≥ 10	27 (14.36)	3 (4.76)	62 (17.92)	
Causes of pelvic pain				< 0.001
Gynecological condition	99 (52.66)	52 (82.54)	275 (79.48)	
Gastrointestinal condition	11 (5.85)	2 (3.17)	3 (0.87)	
Urological condition	5 (2.66)	0 (0)	12 (3.47)	
Myofascial pain	3 (1.6)	0 (0)	4 (1.16)	
Sociopsychological condition	4 (2.13)	2 (3.17)	0 (0)	
Neurological condition	22 (11.7)	1 (1.59)	11 (3.18)	
Others	44 (23.4)	6 (9.52)	41 (11.85)	
Self‐rated pain				< 0.001
1	21 (11.17)	3 (4.76)	12 (3.47)	
2	23 (12.23)	6 (9.52)	54 (15.61)	
3	24 (12.77)	8 (12.7)	36 (10.4)	
4	16 (8.51)	6 (9.52)	37 (10.69)	
5	24 (12.77)	11 (17.46)	64 (18.5)	
6	57 (30.32)	14 (22.22)	58 (16.76)	
7	20 (10.64)	6 (9.52)	56 (16.18)	
8	3 (1.6)	9 (14.29)	29 (8.38)	
Experience of pelvic organ surgery				< 0.001
None	114 (60.64)	24 (38.1)	151 (43.64)	
Gynecological/obstetric surgery	51 (27.13)	36 (57.14)	168 (48.55)	
Urological surgery	6 (3.19)	0 (0)	9 (2.6)	
Colorectal surgery	13 (6.91)	0 (0)	11 (3.18)	
Have undergone surgery, details unclear	4 (2.13)	3 (4.76)	7 (2.02)	
Physical Activity Level Scale				0.014
Low	163 (86.70)	47 (74.60)	269 (77.75)	
Moderate	18 (9.57)	11 (17.46)	67 (19.36)	
High	7 (3.72)	5 (7.94)	10 (2.89)	
Knowledge	6.54 ± 4.01	10.51 ± 7.11	8.24 ± 3.89	< 0.001
Attitude	15.35 ± 2.02	17.16 ± 2.75	16.47 ± 1.83	< 0.001
Practice	13.8 ± 3.02	16.37 ± 4.21	15.69 ± 3.1	< 0.001

Participants demonstrated a mean knowledge score of 7.94 ± 4.52 (possible range: 0–24) (Table 1). Awareness rates, including “Aware” and “Partially aware,” ranged from 33.1% to 82.2%. The highest awareness rate (82.2%) was for the statement that CPP could benefit from complementary therapies such as acupuncture, meditation, traditional Chinese medicine, and yoga (K5d). However, awareness that CPP may relate to musculoskeletal issues was the lowest at 33.1% (K2a), and only 34.2% recognized sexual therapy as a potential treatment for CPP (K5c). In addition, 37.7% acknowledged the role of sociopsychological disorders in CPP onset (K2d) (Table 3).

**TABLE 3:** Responses to knowledge questions.

Knowledge	Aware *N* (%)	Partially aware *N* (%)	Unaware *N* (%)
1. Chronic pelvic pain generally refers to pain originating from pelvic organs and tissues lasting over 6 months, potentially due to tissue damage, inflammation, or nervous system dysfunction	47 (7.9)	327 (54.8)	223 (37.4)
2. Chronic pelvic pain may be associated with the following factors:			
2a. Musculoskeletal disorders	36 (6)	162 (27.1)	399 (66.8)
2b. Visceral organ disorders	28 (4.7)	420 (70.4)	149 (25)
2c. Nervous system disorders	36 (6)	373 (62.5)	188 (31.5)
2d. Sociopsychological disorders	21 (3.5)	204 (34.2)	372 (62.3)
3. Chronic pelvic pain can be treated with nerve blocks, pain medication, botulinum toxin injections, and other methods	38 (6.4)	431 (72.2)	128 (21.4)
4. Chronic pelvic pain may also be treated surgically (e.g., lesion excision or neurolysis, myofascial release surgery)	30 (5)	278 (46.6)	289 (48.4)
5. Chronic pelvic pain can also be treated through the following methods			
5a. Pelvic rehabilitation therapy (e.g., tissue massage, myofascial release, and electrical stimulation)	40 (6.7)	299 (50.1)	258 (43.2)
5b. Psychological and cognitive‐behavioral therapy (e.g., counseling)	27 (4.5)	226 (37.9)	344 (57.6)
5c. Sexual therapy	21 (3.5)	183 (30.7)	393 (65.8)
5d. Other complementary and alternative therapies (e.g., acupuncture, meditation, traditional Chinese medicine, and yoga)	85 (14.2)	406 (68)	106 (17.8)
6. Treatment of pelvic pain requires multidisciplinary collaboration	140 (23.5)	336 (56.3)	121 (20.3)

The mean attitude score was 16.19 ± 2.09 (possible range: 4–20) (Table 1), corresponding to a score rate of 81.0% and indicating an overall positive attitude. Agreement rates for attitude items ranged from 59.3% to 88.0%. The highest agreement rate (88.0%) was for the support of multimodal treatment approaches (A5). The lowest agreement rate (59.3%) was for a preference for nonpharmacological treatments (A4, a descriptive item that was not scored) (Table 4). Regarding illness perception, 23.3% of participants reported feeling a loss of control over symptom impacts during flare‐ups, while 23.1% rated their symptoms as moderately severe (Supporting Table 3).

**TABLE 4:** Responses to attitude questions.

Attitude	Strongly agree *N* (%)	Agree *N* (%)	Neutral *N* (%)	Disagree *N* (%)	Strongly disagree *N* (%)
1. I am hopeful about curing chronic pelvic pain	101 (16.9)	337 (56.4)	152 (25.5)	5 (0.8)	2 (0.3)
2. The treatment for chronic pelvic pain is a long‐term process	148 (24.8)	372 (62.3)	59 (9.9)	16 (2.7)	2 (0.3)
3. Healthcare providers have made sufficient efforts to treat my disease	153 (25.6)	330 (55.3)	98 (16.4)	13 (2.2)	3 (0.5)
4. I am more inclined to receive nonpharmacological treatment	153 (25.6)	201 (33.7)	136 (22.8)	105 (17.6)	2 (0.3)
5. Receiving a combination of various treatments is beneficial for my condition	195 (32.7)	330 (55.3)	62 (10.4)	7 (1.2)	3 (0.5)

The average practice score was 15.17 ± 3.34 (possible range: 5–25) (Table 1). Practice adherence rates ranged from 11.1% to 46.4%. Among the participants, 46.4% actively sought information on CPP from newspapers, media, and other sources (P2). The lowest adherence (11.1%) was observed in regular participation in hospital‐led health education activities (P1). In addition, only 21.0% attended routine follow‐up appointments (P3). Regarding treatment practices, most participants (94.8%) selected physical therapy, such as massage and hot compresses. However, the smallest proportion (36.2%) opted for nerve blocks, and 53.6% practiced meditation (Table 5).

**TABLE 5:** Responses to practice questions.

Practice	Always *N* (%)	Often *N* (%)	Sometimes *N* (%)	Occasionally *N* (%)	Never *N* (%)
1. I regularly participate in health education activities organized by the hospital	16 (2.7)	50 (8.4)	158 (26.5)	286 (47.9)	87 (14.6)
2. I seek knowledge related to my condition through newspapers, media, etc.	138 (23.1)	139 (23.3)	152 (25.5)	127 (21.3)	41 (6.9)
3. I regularly visit the hospital for follow‐up appointments	16 (2.7)	109 (18.3)	291 (48.7)	166 (27.8)	15 (2.5)
4. I fully adhere to the prescribed medication	73 (12.2)	185 (31)	262 (43.9)	72 (12.1)	5 (0.8)
5. I regularly go to the hospital for treatment	28 (4.7)	155 (26)	293 (49.1)	108 (18.1)	13 (2.2)
6. I am willing to try (or have tried) the following treatments:	Yes	No			
6.(1) Tissue massage and myofascial release therapy	324 (54.3)	273 (45.7)			
6.(2) Manual therapy	324 (54.3)	273 (45.7)			
6.(3) Nerve blocks	216 (36.2)	381 (63.8)			
6.(4) Physical therapy (e.g., massage, hot compresses)	566 (94.8)	31 (5.2)			
6.(5) Pelvic floor training	409 (68.5)	188 (31.5)			
6.(6) Pelvic muscle stretching	394 (66)	203 (34)			
6.(7) Psychological counseling	359 (60.1)	238 (39.9)			
6.(8) Meditation	320 (53.6)	277 (46.4)			
6.(9) Traditional Chinese medicine	441 (73.9)	156 (26.1)			
6.(10) Acupuncture	332 (55.6)	265 (44.4)			
6.(11) Yoga	448 (75)	149 (25)			
6.(12) Tai Chi	418 (70)	179 (30)			

As for the KAP comparison between B‐IPQ classes, participants with high illness perception scored significantly higher in knowledge (*p* < 0.001), attitude (*p* < 0.001), and practice (*p* < 0.001) (Table 2). The attitude score rates were 76.7%, 85.8%, and 82.3% for the low, high, and medium illness perception classes, respectively. At the item level, all groups reported the lowest scores in “feeling hopeful about curing chronic pelvic pain” (A1) and “regularly participating in hospital‐based health education activities” (P1). Notably, participants with high illness perception scored the lowest on understanding sexual therapy as a treatment modality (K5.3), while those with low illness perception had the lowest score for identifying musculoskeletal disorders as a potential cause of CPP (K2.1) (Supporting Table 4).

Correlation analysis revealed significant positive correlations between knowledge and attitude (*r* = 0.363, *p* < 0.001), between knowledge and practice (*r* = 0.401, *p* < 0.001), and between attitude and practice (*r* = 0.268, *p* < 0.001) (Supporting Table 5). Path analysis using observed total scores further confirmed the direct associations: knowledge was directly associated with attitude (*β* = 0.394, 95% CI: 0.329 to 0.459, *p* < 0.001) and practice (*β* = 0.378, 95% CI: 0.307 to 0.449, *p* < 0.001), and attitude was also directly associated with practice (*β* = 0.148, 95% CI: 0.071 to 0.225, *p* < 0.001). In addition, knowledge demonstrated a significant total association with practice (*β* = 0.436, 95% CI: 0.375 to 0.498, *p* < 0.001), with an indirect association mediated through attitude (*β* = 0.058, 95% CI: 0.026 to 0.090, *p* < 0.001) (Figure 1 and Table 6). The indirect pathway, although statistically significant, accounted for a relatively modest proportion of the total association, suggesting that knowledge was predominantly associated with practice through the direct pathway.

**FIGURE 1:**
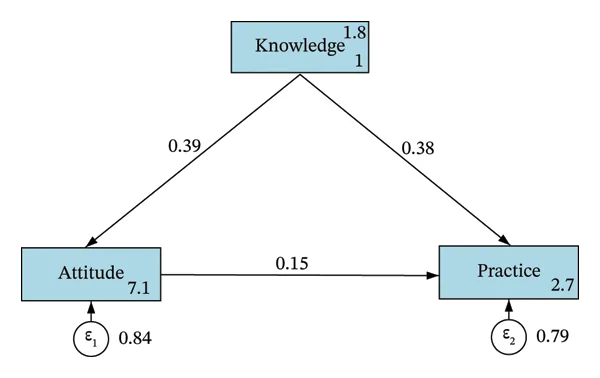
Path analysis model showing the associations between KAP scores. Knowledge, attitude, and practice were modeled as observed total scores. Direction of association is indicated by arrows. The standardized path coefficients are presented alongside the arrows.

**TABLE 6:** Direct and indirect associations from path analysis using observed total scores.

Model paths	Total associations		Direct associations		Indirect associations	
	*β* (95% CI)	*p*	*β* (95% CI)	*p*	*β* (95% CI)	*p*
Knowledge ⟶ Attitude	0.394 (0.329, 0.459)	< 0.001	0.394 (0.329, 0.459)	< 0.001		
Knowledge ⟶ Practice	0.436 (0.375, 0.498)	< 0.001	0.378 (0.307, 0.449)	< 0.001	0.058 (0.026, 0.090)	< 0.001
Attitude ⟶ Practice	0.148 (0.071, 0.225)	< 0.001	0.148 (0.071, 0.225)	< 0.001		

To identify factors associated with good practice (defined as a practice score ≥ 60% of the total, i.e., ≥ 15 points), univariate and multivariate logistic regression analyses were performed (Table 7). Variables with *p* < 0.05 in the univariate analysis were entered into the multivariate model. Collinearity diagnostics confirmed no multicollinearity among included variables (all VIFs < 2.0). In the multivariate model, higher knowledge scores (OR = 1.107, 95% CI: 1.044–1.173, *p* = 0.001) and higher attitude scores (OR = 1.161, 95% CI: 1.039–1.297, *p* = 0.009) were independently associated with good practice. Compared with the low illness perception class (Class 1), participants in the medium illness perception class (Class 3) were significantly more likely to report good practice (OR = 2.148, 95% CI: 1.338–3.448, *p* = 0.002). Participants with a BMI ≥ 28.0 kg/m^2^ had significantly lower odds of good practice compared with those with a BMI < 18.5 (OR = 0.123, 95% CI: 0.030–0.511, *p* = 0.004). Having one pregnancy (OR = 3.728, *p* = 0.015) or two pregnancies (OR = 3.331, *p* = 0.033) was associated with higher odds of good practice relative to nulligravida. Lack of experience with pelvic floor muscle exercises after childbirth was negatively associated with good practice (OR = 0.461, 95% CI: 0.244–0.870, *p* = 0.017). Having a neurological condition as the perceived cause of pelvic pain was associated with lower odds of good practice compared with gynecological conditions (OR = 0.363, 95% CI: 0.143–0.922, *p* = 0.033). In addition, a monthly income of 5000–10,000 Chinese yuan was associated with higher odds of good practice relative to income < 2000 (OR = 3.443, 95% CI: 1.120–10.587, *p* = 0.031).

**TABLE 7:** Univariate and multivariate logistic regression analysis of factors associated with good practice.

Variables	Univariate logistic regression		Multivariate logistic regression	
	OR (95% CI)	*p*	OR (95% CI)	*p*
Knowledge	1.157 (1.106, 1.211)	< 0.001	1.107 (1.044, 1.173)	0.001
Attitude	1.244 (1.143, 1.355)	< 0.001	1.161 (1.039, 1.297)	0.009
Classes of B‐IPQ				
Class 1 (ref)	—	—	—	—
Class 2	2.628 (1.452, 4.759)	< 0.001	1.674 (0.760, 3.691)	0.201
Class 3	2.946 (2.041, 4.254)	< 0.001	2.148 (1.338, 3.448)	0.002
Age, years				
< 35 (ref)	—	—		
35–44	1.552 (0.901, 2.674)	0.113		
45–54	1.016 (0.597, 1.731)	0.953		
≥ 55	0.630 (0.364, 1.092)	0.100		
BMI (kg/m^2^)				
< 18.5 (ref)	—	—	—	—
18.50–23.99	0.691 (0.277, 1.724)	0.428	0.409 (0.124, 1.348)	0.142
24.00–27.99	0.631 (0.248, 1.606)	0.334	0.450 (0.134, 1.511)	0.196
≥ 28.0	0.228 (0.075, 0.692)	0.009	0.123 (0.030, 0.511)	0.004
Marital status				
Married (ref)	—	—		
Single	0.793 (0.349, 1.802)	0.579		
Divorced	0.623 (0.287, 1.351)	0.231		
Other	1.342 (0.121, 14.886)	0.811		
Gravidity				
0 (ref)	—	—	—	—
1	3.625 (1.497, 8.779)	0.004	3.728 (1.288, 10.787)	0.015
2	2.766 (1.137, 6.730)	0.025	3.331 (1.105, 10.040)	0.033
3	2.000 (0.721, 5.546)	0.183	1.942 (0.553, 6.825)	0.301
> 3	2.429 (0.805, 7.331)	0.115	2.560 (0.652, 10.060)	0.178
Parity				
0 (ref)	—	—		
1	1.924 (0.963, 3.842)	0.064		
2	1.547 (0.713, 3.356)	0.270		
3	0.432 (0.115, 1.622)	0.214		
Experience of cesarean section				
Yes (ref)	—	—		
No	0.831 (0.589, 1.174)	0.294		
Experience of pelvic floor muscle exercises after childbirth				
Yes (ref)	—	—	—	—
No	0.202 (0.120, 0.340)	< 0.001	0.461 (0.244, 0.870)	0.017
Residence				
Urban (ref)	—	—	—	—
Rural	0.362 (0.230, 0.570)	< 0.001	0.709 (0.366, 1.373)	0.307
Suburban	0.308 (0.196, 0.486)	< 0.001	0.621 (0.334, 1.157)	0.133
Average commute time to the hospital, hours				
< 1 (ref)	—	—	—	—
1.00–1.99	1.329 (0.892, 1.978)	0.162	1.275 (0.775, 2.097)	0.339
2.00–2.99	0.580 (0.354, 0.951)	0.031	1.519 (0.737, 3.134)	0.258
≥ 3	0.699 (0.343, 1.422)	0.323	1.512 (0.585, 3.909)	0.393
Ethnicity				
Han (ref)	—	—		
Minority ethnic group	0.845 (0.436, 1.636)	0.617		
Religious belief				
Yes (ref)	—	—		
No	1.266 (0.749, 2.139)	0.379		
Education				
Primary school or below (ref)	—	—	—	—
Middle school/high school/technical school	1.432 (0.604, 3.398)	0.415	1.462 (0.511, 4.180)	0.479
Associate/bachelor’s degree	3.675 (1.572, 8.595)	0.003	3.074 (0.986, 9.581)	0.053
Master’s degree or above	4.667 (1.718, 12.676)	0.003	2.110 (0.526, 8.468)	0.292
Physically demanding occupations				
Yes (ref)	—	—	—	—
No	2.651 (1.295, 5.424)	0.008	1.118 (0.414, 3.015)	0.826
Unsure	1.654 (0.734, 3.725)	0.225	2.408 (0.859, 6.749)	0.095
Monthly income, Chinese yuan				
< 2000 (ref)	—	—	—	—
2000–5000	2.728 (1.171, 6.352)	0.020	1.770 (0.631, 4.967)	0.278
5000–10,000	9.720 (4.052, 23.319)	< 0.001	3.443 (1.120, 10.587)	0.031
10,000–20,000	3.920 (1.280, 12.006)	0.017	1.362 (0.328, 5.650)	0.670
> 20,000	3.450 (1.079, 11.033)	0.037	0.665 (0.123, 3.593)	0.635
Prefer not to disclose	4.558 (1.863, 11.148)	0.001	2.567 (0.837, 7.873)	0.099
Duration of pelvic pain, years				
< 2 (ref)	—	—		
2.00–4.99	0.984 (0.631, 1.535)	0.944		
5.00–9.99	0.771 (0.476, 1.250)	0.291		
≥ 10	0.854 (0.494, 1.474)	0.571		
Causes of pelvic pain				
Gynecological condition (ref)	—	—	—	—
Gastrointestinal condition	1.064 (0.380, 2.983)	0.906	2.388 (0.662, 8.616)	0.184
Urological condition	0.912 (0.341, 2.443)	0.855	1.374 (0.416, 4.541)	0.602
Myofascial pain	1.596 (0.306, 8.322)	0.579	1.499 (0.249, 9.037)	0.659
Sociopsychological condition	1.277 (0.231, 7.050)	0.779	2.420 (0.232, 25.295)	0.460
Neurological condition	0.395 (0.193, 0.811)	0.011	0.363 (0.143, 0.922)	0.033
Others	0.814 (0.515, 1.286)	0.378	0.767 (0.421, 1.398)	0.387
Self‐rated pain				
1 (ref)	—	—	—	—
2	1.828 (0.828, 4.036)	0.136	1.633 (0.624, 4.270)	0.317
3	1.575 (0.696, 3.562)	0.275	2.022 (0.753, 5.431)	0.162
4	2.533 (1.082, 5.929)	0.032	2.959 (1.006, 8.698)	0.049
5	1.750 (0.809, 3.788)	0.155	2.242 (0.855, 5.879)	0.101
6	2.008 (0.949, 4.249)	0.068	2.045 (0.792, 5.280)	0.139
7	3.383 (1.497, 7.649)	0.003	2.256 (0.806, 6.316)	0.121
8	3.015 (1.185, 7.671)	0.021	1.844 (0.560, 6.070)	0.314
Experience of pelvic organ surgery				
None (ref)	—	—		
Gynecological/obstetric surgery	1.141 (0.811, 1.605)	0.450		
Urological surgery	3.092 (0.854, 11.192)	0.085		
Colorectal surgery	1.877 (0.755, 4.666)	0.175		
Have undergone surgery, details unclear	1.391 (0.455, 4.255)	0.562		

*Note:* Multivariate model included variables with *p* < 0.05 in univariate analysis. Empty cells in the multivariate column indicate that the variable was not included in the multivariate model.

A sensitivity analysis using raw practice scores as a continuous outcome in linear regression was performed to examine the robustness of the dichotomized findings (Supporting Table 6). The multivariable model explained 40.5% of the variance in practice scores (*R* ^2^ = 0.405). Consistent with the logistic regression results, higher knowledge scores (*β* = 0.244, *p* < 0.001), higher attitude scores (*β* = 0.213, *p* < 0.001), lack of pelvic floor muscle exercise experience (*β* = −1.100, *p* < 0.001), BMI ≥ 28.0 kg/m^2^ (*β* = −2.016, *p* = 0.007), having one pregnancy (*β* = 1.802, *p* = 0.003) or two pregnancies (*β* = 1.873, *p* = 0.003), and monthly income of 5000–10,000 yuan (*β* = 1.437, *p* = 0.019) remained independently associated with practice scores. Notably, unlike the logistic regression in which only Class 3 (medium illness perception) was independently associated with good practice, the linear regression revealed that both Class 2 (high illness perception; *β* = 1.257, *p* = 0.006) and Class 3 (medium illness perception; *β* = 1.085, *p* < 0.001) were independently associated with higher practice scores compared with Class 1 (low illness perception).

## 4. Discussion

Female outpatients demonstrated poor knowledge, positive attitudes, and moderate practices toward CPP. Participants with high illness perception recorded the highest B‐IPQ and KAP scores. KAP scores showed positive correlations and direct associations, with knowledge exerting an indirect association with practice through attitude.

The finding of poor knowledge reflects a persistent clinical challenge: CPP is often misconstrued as purely gynecological, which may be associated with delays in multidisciplinary management. In contrast, the positive attitudes observed indicate a willingness to seek and adhere to treatment, although the moderate practice scores suggest that this willingness has not yet been fully translated into self‐care. Overall compliance with recommended practices remains suboptimal [22], underscoring the need for patient‐centered interventions that emphasize self‐care guidance.

In the knowledge dimension, awareness of musculoskeletal contributions to CPP remained low, reflecting the prevailing view that CPP is primarily gynecological. Evidence increasingly implicates factors such as pelvic floor muscle tension, myofascial pain, and posture‐related dysfunction [23]. As musculoskeletal disorders are often underdiagnosed [24], this poor awareness may be associated with delayed effective management. Our finding that low knowledge was associated with lower practice scores is consistent with evidence that subgroups of CPP syndromes share a common pronociceptive pain modulation profile interrelated with augmented clinical pain [12], and that negative illness perceptions are linked to a pronociceptive modulation profile and increased pain severity [11]. These findings collectively suggest that inadequate knowledge may perpetuate maladaptive illness perceptions, which in turn are associated with unfavorable pain processing and poorer self‐management. Knowledge of sexual therapy and the role of sociopsychological disorders was similarly limited, possibly due to sociocultural barriers or stigma [25, 26]. Given the high economic burden of CPP [27], integrating information on these factors into patient education is crucial to broaden understanding, reduce stigma, and encourage engagement with a wider range of evidence‐based treatments.

In the attitude dimension, more than half of the participants held positive attitudes toward nonpharmacological treatments. Aligning with these results, the efficacy of physical therapy, acupuncture, and mindfulness techniques has been demonstrated in CPP management [28]. The positive attitudes observed in our study are further supported by evidence that myofascial physical therapy produces improvements spanning anatomical, neurophysiological, and psychological domains in women with CPP syndrome [13], providing a mechanistic basis for patients’ favorable perceptions of nonpharmacological approaches. However, there remains room for fostering positive attitudes, suggesting the need for education on the benefits and mechanisms of nonpharmacological options. Furthermore, almost a quarter of participants reported a loss of control over their symptoms, partly due to the unpredictable nature of CPP exacerbations. Interventions focusing on pain self‐management techniques, such as cognitive behavioral therapy (CBT) and mindfulness practices, may be effective in empowering patients and improving their perceived control over symptoms [29].

The low participation in hospital‐led health education suggests barriers to accessing or engaging with formal programs. Prior studies identify logistical constraints, lack of personalized content, and limited perceived relevance as deterrents [30]. Addressing these barriers through flexible, patient‐centered platforms and telemedicine could enhance engagement and continuity of care [31]. In China, patient‐facing digital health resources for CPP and related conditions are increasingly available, but their development remains fragmented. Internet hospitals and telemedicine services have expanded substantially in recent years [32], while published evidence on digital interventions for pelvic pain–related conditions in China remains limited and has mainly focused on hospital‐ or research‐based online nursing and management interventions rather than established patient‐led communities [33]. Regional patient education and support initiatives, including hospital‐led online patient groups, have also been reported for related pelvic pain conditions such as endometriosis. However, to our knowledge, there is still no nationwide, dedicated, CPP‐specific patient organization or comprehensive patient‐centered Chinese‐language e‐health platform comparable to some resources developed internationally [34]. Developing such platforms with multidisciplinary clinical oversight, patient involvement, and opportunities for peer‐to‐peer interaction may provide a low‐threshold complement to formal hospital‐based education, consistent with its noninvasive nature and efficacy [35]. In contrast, adherence to nerve blocks remained low, likely due to safety concerns, invasiveness, and limited patient awareness [36]. Comprehensive counseling on the benefits of such interventions and correction of misconceptions could improve uptake.

Patients exhibiting heightened illness perception demonstrated an “engaged threat” profile [37]. They reported acute awareness, severe symptoms, and emotional distress but also high confidence in treatment and personal control. It should be noted that, because LPA was conducted on raw (nonreversed) B‐IPQ item scores, higher scores on items 3 (personal control), 4 (treatment control), and 7 (understanding) directly indicated greater perceived control and illness comprehension. Therefore, the high illness perception class genuinely exhibited both the greatest perceived threat and the strongest sense of personal and treatment control. This supports Leventhal’s Common‐Sense Model: a high threat perception, when combined with a belief in actionable solutions, stimulates positive coping. Consequently, this class, despite being associated with more severe pain (scores ≥ 5), a higher prevalence of prior pelvic organ surgery, and lower physical activity, demonstrated the highest KAP scores. Clinically, interventions for this group should focus on reinforcing their perceptions of personal and treatment control, including proactive discussion of sexual health, an aspect consistently underaddressed in CPP consultations, rather than attempting to reduce their often realistic threat perception [38].

In contrast, participants with low illness perception demonstrated limited perceived illness threat and weaker illness coherence, and were more likely to belong to minority ethnic groups or hold religious beliefs. This profile warrants specific clinical attention. Religious beliefs can influence pain perception, coping strategies, and willingness to engage with biomedical treatments, with research indicating that such beliefs may shape health‐seeking behaviors by promoting acceptance of suffering or prioritizing spiritual over clinical interventions [39]. For this subgroup, clinicians should adopt a culturally sensitive approach: acknowledging spiritual frameworks as a valid coping resource while systematically bridging gaps in biomedical understanding. Strategies may include deploying community health workers or peer educators from minority groups, using bilingual or culturally adapted educational materials, and framing CPP education within culturally resonant narratives [40]. Their lowest recognition of musculoskeletal disorders as a CPP cause further suggests that educational content should explicitly address the biopsychosocial etiology of CPP in accessible, nonstigmatizing language.

As for participants with medium illness perception (Class 3), their moderate self‐care engagement despite incomplete illness understanding suggests a clinically actionable profile. For this group, targeted interventions aimed at deepening illness coherence and reinforcing self‐efficacy, such as structured group education programs or brief motivational counseling, may effectively elevate practice to a higher level.

Our path analysis confirmed positive, direct associations among KAP scores. The clinical implication is twofold. First, the strong link from knowledge directly to practice underscores the necessity of patient education focused on core CPP knowledge. Second, the significant indirect pathway demonstrates that education alone may be insufficient. To maximize practice adherence, interventions must also include strategies specifically designed to cultivate positive attitudes. Consistent with prior findings in other health conditions [41], improving patients’ understanding of CPP (knowledge) is key to positively shaping their outlook and emotional response (attitude), which in turn is associated with adherence to recommended self‐management behaviors (practice). However, the indirect association through attitude was relatively modest (*β* = 0.058), suggesting that while attitude partially mediates the knowledge–practice relationship, the predominant pathway is the direct association between knowledge and practice. This finding implies that in the context of CPP, knowledge acquisition may translate into behavioral change through mechanisms beyond attitudinal shifts alone, such as enhanced self‐efficacy or improved illness coherence.

The logistic regression analysis identified independent predictors of good practice. Higher knowledge and attitude scores were independently associated with good practice, reinforcing the path analysis findings. Notably, the medium illness perception class (Class 3), rather than the high illness perception class (Class 2), was significantly associated with good practice in the adjusted model. This aligns with the extended Common‐Sense Model, which posits that threat perceptions motivate adaptive coping only when paired with sufficient knowledge and confidence [42]. The attenuation of the association between the high illness perception class and good practice in the multivariable model likely reflects the substantial overlap between this class and covariates already included in the model, such as pain severity, prior pelvic surgery, and healthcare exposure, which may have absorbed much of the explanatory variance attributable to high illness perception. In addition, the relatively small size of Class 2 (*n* = 63, 10.55%) may have limited the statistical power to detect an independent association in the fully adjusted model. Importantly, the sensitivity analysis using continuous practice scores revealed that both Class 2 (*β* = 1.257, *p* = 0.006) and Class 3 (*β* = 1.085, *p* < 0.001) were independently associated with higher practice scores, suggesting that the dichotomization of practice scores in the logistic regression may have contributed to the loss of significance for Class 2 by reducing statistical information.

Among the sociodemographic predictors, having one or two pregnancies compared with nulligravidity was independently associated with good practice. Although seemingly counterintuitive, this finding may reflect the increased exposure to the healthcare system during antenatal and postnatal care. Women with prior pregnancies are more likely to have had structured contact with obstetric and gynecological services, receiving related health education and pelvic floor guidance, which may translate into more proactive self‐management behaviors in the context of CPP [43]. Regarding income, the observation that the 5000 to 10,000 yuan group demonstrated better practice than higher‐income groups warrants consideration. While higher income generally facilitates healthcare access, very high earners may face competing occupational and time demands that reduce engagement with self‐management routines. In addition, individuals in the middle‐income bracket may be more motivated to optimize cost‐effective nonpharmacological strategies, given that out‐of‐pocket expenditure for chronic pain management remains substantial in China [44, 45].

Lack of pelvic floor muscle exercise experience after childbirth and higher BMI (≥ 28.0 kg/m^2^) were negatively associated with good practice, underscoring the need for postpartum rehabilitation counseling and targeted guidance for patients with obesity. The association between neurological conditions and lower odds of good practice likely reflects the complexity and treatment resistance of neuropathic pelvic pain, which involves central sensitization and requires specialized interdisciplinary management [46]. These findings provide empirical evidence for the formulation of targeted health policies and resource allocation strategies. In clinical settings, KAP analysis can help optimize patient education programs by identifying specific knowledge gaps and attitudinal barriers that require intervention [8].

This study has several limitations. First, medical insurance status was not collected in this survey. In China, basic medical insurance covers approximately 95% of the population as of 2023, yet out‐of‐pocket payments remain substantial for chronic disease management [44, 45]. Insurance coverage may influence KAP by improving healthcare access and reducing financial barriers to treatment adherence [47]. Combined with the significant association between income and KAP scores observed in this study, the interaction between insurance and economic factors warrants future investigation. Second, the reliance on self‐reported data may have introduced social desirability bias, potentially inflating KAP scores [48]. Third, data collection was restricted to a single center, which may limit the generalizability of the findings. Fourth, the cross‐sectional design permitted only the observation of associations among KAP dimensions, rather than establishing causal relationships. Although path analysis was employed to examine hypothesized directional associations guided by the KAP theoretical framework, this approach provides statistical inference rather than definitive causal evidence. Longitudinal studies are needed to establish the temporal sequence among KAP. Fifth, the self‐designed KAP questionnaire demonstrated acceptable overall reliability (Cronbach’s *α* = 0.869), but the attitude dimension showed relatively lower internal consistency (*α* = 0.726), which may have attenuated the observed associations involving attitude scores. This may partly account for the relatively modest indirect effect of knowledge on practice through attitude observed in the path analysis. Furthermore, the questionnaire development process lacks comprehensive construct validity testing and the content validity was preliminary. Future studies should consider further refining the attitude scale or adopting validated instruments to improve measurement precision. Finally, convenience sampling could lead to selection bias, thus limiting the representativeness of the study population.

In conclusion, female outpatients exhibited poor knowledge, positive attitudes, and moderate practices regarding CPP. Participants with heightened illness perceptions demonstrated elevated KAP scores. Knowledge, attitude, illness perception profile, and postpartum pelvic floor exercise experience were identified as independent predictors of good practice, offering specific targets for clinical intervention. Three distinct illness perception profiles were identified, each warranting tailored intervention strategies: reinforcing perceived control for the high‐perception group, culturally adapted education for the low‐perception group, and structured knowledge‐building for the medium‐perception group. Given the direct associations among KAP dimensions, tailored educational interventions are recommended to enhance knowledge related to CPP, foster supportive attitudes, and improve adherence to treatment practices. These targeted efforts can better equip patients to manage CPP and adopt beneficial health practices, thereby improving clinical outcomes.

## Author Contributions

Yiran Qiu and Yiming Wang carried out the studies, participated in collecting data, performed the statistical analysis, and drafted the manuscript. Fan Li, Guangting Mao, Chubin Xing, and Xiaoyu Wang participated in acquisition, analysis, or interpretation of data.

## Funding

No funding was received for this research.

## Disclosure

All authors read and approved the final manuscript.

## Ethics Statement

The study was approved by the Ethics Committee of the First Hospital of Jilin University (24K220‐001).

## Consent

All participants were informed about the study protocol and provided written informed consent to participate in the study. The authors confirm that all methods were performed in accordance with the relevant guidelines. All procedures were performed in accordance with the ethical standards laid down in the 1964 Declaration of Helsinki and its later amendments.

## Conflicts of Interest

The authors declare no conflicts of interest.

## Supporting Information

Additional supporting information can be found online in the Supporting Information section.

## Supporting information


**Supporting Information 1** Supporting Figure 1. The score distribution of three groups across Brief Illness Perception Questionnaire (B‐IPQ) items. Raw (nonreversed) item scores were used in the latent profile analysis. Higher scores on items 3 (personal control), 4 (treatment control), and 7 (understanding) indicate greater perceived control or comprehension, whereas higher scores on the remaining items indicate greater perceived illness threat or emotional distress. Class 1 = low illness perception (31.49%); Class 2 = high illness perception (10.55%); and Class 3 = medium illness perception (57.96%).

**Supporting Information 2** Supporting Table 1. Physical activity level of participants. Supporting Table 2. The goodness of fit regarding clustering models based on varying numbers of class. Supporting Table 3. Participants’ responses to illness perception. Supporting Table 4. The distribution of KAP scores among three Brief Illness Perception Questionnaire (B‐IPQ) groups (attitude item A4 was descriptive only and was not scored). Supporting Table 5. Spearman correlation analysis of KAP scores. Supporting Table 6. Sensitivity analysis: multivariable linear regression of factors associated with practice scores (continuous outcome).

## Data Availability

All data generated or analyzed during this study are included in this published article.
