# Supplementary material for: Knowledge, Attitudes, Practices, and Illness Perception of Female Outpatients With Chronic Pelvic Pain
Source: Pain Res Manag. 2026 Oct 5;2026:5017608. doi: 10.1155/prm/5017608 (PMC13639545; doi:10.1155/prm/5017608)
Supplement: Supplementary file 1 [file PRM-2026-5017608-s002.docx]

**Supplementary Table 1. Physical activity level of participants**

| **Physical Activity Level Scale table** | N (%) |
| --- | --- |
| **1.** **What is the intensity of your physical exercise?** |  |
| a. Light activity (e.g., walking, calisthenics, croquet) | 250(41.9%) |
| b. Low-intensity, moderate activity (e.g., recreational sports, jogging, tai chi) | 230(38.5%) |
| c. Moderate-intensity, sustained activity (e.g., cycling, running, table tennis) | 94(15.7%) |
| d. High-intensity, short-duration activity with rapid breathing and heavy sweating (e.g., badminton, basketball) | 11(1.8%) |
| e. High-intensity, sustained activity with rapid breathing and heavy sweating (e.g., competitive running, aerobics routines, swimming) | 12(2.0%) |
| **2.** **How many minutes do you spend on the above physical activities in a single session?** |  |
| a. Less than 10 minutes | 30(5.0%) |
| b.11-20 minutes | 164(27.5%) |
| c.21-30 minutes | 289(48.4%) |
| d.31-59 minutes | 87(14.6%) |
| e. 60 minutes or more | 27(4.5%) |
| **3.** **How frequently do you engage in the above physical activities each month?** |  |
| a. Less than once a month | 27(4.5%) |
| b. 2-3 times a month | 116(19.4%) |
| c. 1-2 times a week | 261(43.7%) |
| d. 3-5 times a week | 140(23.5%) |
| e. About once a day | 53(8.9%) |

**Supplementary Table 2. The goodness of fit regarding clustering models based on varying numbers of class.**

| **Classes** | **LogLik** | **AIC** | **BIC** | **adjusted BIC** | **ICL** | **Entropy** | **prob_min** | **prob_max** | **n_min** | **n_max** | **BLRT** | **BLRT_p** |
| --- | --- | --- | --- | --- | --- | --- | --- | --- | --- | --- | --- | --- |
| 1 | -4966.95 | 9945.90 | 9972.25 | 9953.20 | -9972.25 | 1.00 | 1.00 | 1.00 | 1.00 | 1.00 | NA | NA |
| 2 | -4858.27 | 9736.54 | 9780.46 | 9748.71 | -9974.63 | 0.57 | 0.80 | 0.91 | 0.38 | 0.62 | 217.35 | 0.01 |
| 3 | -4796.17 | 9620.35 | 9681.84 | 9637.39 | -9815.94 | 0.79 | 0.86 | 0.92 | 0.11 | 0.58 | 124.19 | 0.01 |
| 4 | -4771.27 | 9578.54 | 9657.59 | 9600.45 | -9886.36 | 0.72 | 0.82 | 0.88 | 0.11 | 0.34 | 49.81 | 0.01 |
| 5 | -4769.87 | 9583.75 | 9680.37 | 9610.53 | -10026.49 | 0.64 | 0.07 | 0.89 | 0.02 | 0.33 | 2.79 | 0.44 |

Abbreviations: LogLik, Log-Likelihood; AIC, Akaike information criterion; BIC, Bayesian information criterion; ICL, integrated completed likelihood; BLRT, bootstrapped likelihood ratio test; prob_min, minimum average posterior class-membership probability; prob_max, maximum average posterior class-membership probability

**Supplementary Table 3. Participants’ responses to illness perception**

| Illness Perception | 0 | 1 | 2 | 3 | 4 | 5 | 6 | 7 | 8 | 9 | 10 |
| --- | --- | --- | --- | --- | --- | --- | --- | --- | --- | --- | --- |
| 1. To what extent does your disease impact your life? (0 represents no impact, 10 represents a severe impact) | 7(1.2%) | 56(9.4%) | 75(12.6%) | 55(9.2%) | 137(22.9%) | 109(18.3%) | 94(15.7%) | 37(6.2%) | 25(4.2%) | 2(0.3%) | 0 (0%) |
| 2. How long do you think your disease will last? (0 represents a very short time, 10 represents forever) | 17(2.8%) | 105(17.6%) | 113(18.9%) | 95(15.9%) | 86(14.4%) | 98(16.4%) | 40(6.7%) | 12(2%) | 17(2.8%) | 3(0.5%) | 11(1.8%) |
| 3. When your disease flares up, how much control do you feel you have over its impact? (0 represents no control, 10 represents maximum control) | 5(0.8%) | 139(23.3%) | 110(18.4%) | 48(8%) | 45(7.5%) | 51(8.5%) | 73(12.2%) | 50(8.4%) | 52(8.7%) | 12(2%) | 12(2%) |
| 4. To what extent do you feel your treatment helps with your disease? (0 represents no help, 10 represents extreme help) | 6(1%) | 14(2.3%) | 19(3.2%) | 33(5.5%) | 49(8.2%) | 106(17.8%) | 124(20.8%) | 72(12.1%) | 76(12.7%) | 46(7.7%) | 52(8.7%) |
| 5. How severe are your symptoms? (0 represents no symptoms, 10 represents very severe symptoms) | 1(0.2%) | 40(6.7%) | 94(15.7%) | 94(15.7%) | 138(23.1%) | 85(14.2%) | 77(12.9%) | 40(6.7%) | 27(4.5%) | 1(0.2%) | 0 (0%) |
| 6. How concerned are you about your disease? (0 represents not at all concerned, 10 represents extremely concerned) | 4(0.7%) | 11(1.8%) | 46(7.7%) | 30(5%) | 92(15.4%) | 81(13.6%) | 132(22.1%) | 50(8.4%) | 67(11.2%) | 33(5.5%) | 51(8.5%) |
| 7. To what extent do you feel you understand your condition? (0 represents no understanding, 10 represents full understanding) | 8(1.3%) | 22(3.7%) | 113(18.9%) | 101(16.9%) | 59(9.9%) | 86(14.4%) | 72(12.1%) | 44(7.4%) | 59(9.9%) | 13(2.2%) | 20(3.4%) |
| 8. To what extent does your disease impact your emotions? (0 represents no emotional impact, 10 represents extreme emotional impact) | 16(2.7%) | 27(4.5%) | 85(14.2%) | 82(13.7%) | 112(18.8%) | 97(16.2%) | 66(11.1%) | 46(7.7%) | 29(4.9%) | 17(2.8%) | 20(3.4%) |

**Supplementary Table 4. The distribution of KAP scores among three Brief Illness Perception Questionnaire (B-IPQ) groups**

|  | **Group1** | **Group2** | **Group3** |
| --- | --- | --- | --- |
| K1 | 0.48 | 0.86 | 0.80 |
| K2.1 | **0.30** | 0.86 | **0.36** |
| K2.2 | 0.76 | 0.90 | 0.80 |
| K2.3 | 0.80 | 0.86 | 0.70 |
| K2.4 | 0.32 | 0.75 | 0.40 |
| K3 | 0.69 | 1.06 | 0.90 |
| K4 | 0.31 | 0.94 | 0.64 |
| K5.1 | 0.41 | 1.03 | 0.68 |
| K5.2 | 0.35 | 0.83 | 0.47 |
| K5.3 | 0.32 | **0.56** | 0.38 |
| K5.4 | 0.91 | 0.89 | 1.01 |
| K6 | 0.89 | 0.98 | 1.12 |
| A1 | **3.68** | **4.14** | **3.96** |
| A2 | 3.95 | 4.33 | 4.11 |
| A3 | 3.79 | 4.29 | 4.12 |
| A5 | 3.94 | 4.40 | 4.28 |
| P1 | **2.02** | **3.08** | **2.43** |
| P2 | 3.34 | 3.24 | 3.37 |
| P3 | 2.59 | 3.19 | 3.03 |
| P4 | 3.05 | 3.60 | 3.58 |
| P5 | 2.81 | 3.25 | 3.28 |

The lowest KAP scores in each group were in bold.

**Supplementary Table 5. Spearman correlation analysis of KAP scores**

|  | **Knowledge** | **Attitude** | **Practice** |
| --- | --- | --- | --- |
| **Knowledge** | 1.000 |  |  |
| **Attitude** | 0.363 (P<0.001) | 1.000 |  |
| **Practice** | 0.401 (P<0.001) | 0.268 (P<0.001) | 1.000 |

**Supplementary Table 6. Sensitivity analysis: univariate and multivariate linear regression of factors associated with practice scores (continuous outcome).**

| **Variable / category** | **Univariate β (95% CI)** | **P** | **Multivariable β (95% CI)** | **P** |
| --- | --- | --- | --- | --- |
| **Knowledge** | 0.323 (0.269, 0.376) | <0.001 | 0.244 (0.182, 0.306) | <0.001 |
| **Attitude** | 0.474 (0.351, 0.596) | <0.001 | 0.213 (0.091, 0.334) | <0.001 |
| **Classes of B-IPQ** |  |  |  |  |
| Class 1 (ref) | — | — | — | — |
| Class 2 | 2.567 (1.651, 3.483) | <0.001 | 1.257 (0.361, 2.154) | 0.006 |
| Class 3 | 1.893 (1.323, 2.463) | <0.001 | 1.085 (0.529, 1.641) | <0.001 |
| **BMI, kg/m²** |  |  |  |  |
| <18.5 (ref) | — | — | — | — |
| 18.50–23.99 | 0.057 (−1.347, 1.461) | 0.937 | −0.758 (−1.952, 0.436) | 0.213 |
| 24.00–27.99 | −0.080 (−1.518, 1.359) | 0.914 | −0.513 (−1.735, 0.709) | 0.411 |
| ≥28.0 | −1.603 (−3.325, 0.120) | 0.068 | −2.016 (−3.481, −0.551) | 0.007 |
| **Gravidity** |  |  |  |  |
| 0 (ref) | — | — | — | — |
| 1 | 2.644 (1.265, 4.024) | <0.001 | 1.802 (0.616, 2.989) | 0.003 |
| 2 | 2.439 (1.048, 3.830) | <0.001 | 1.873 (0.651, 3.096) | 0.003 |
| 3 | 1.625 (0.006, 3.244) | 0.049 | 0.906 (−0.524, 2.337) | 0.214 |
| >3 | 1.747 (−0.013, 3.508) | 0.052 | 0.722 (−0.824, 2.267) | 0.360 |
| **Experience of pelvic floor muscle exercises after childbirth** |  |  |  |  |
| Yes (ref) | — | — | — | — |
| No | −2.638 (−3.266, −2.009) | <0.001 | −1.100 (−1.737, −0.464) | <0.001 |
| **Residence** |  |  |  |  |
| Urban (ref) | — | — | — | — |
| Rural | −1.321 (−2.041, −0.602) | <0.001 | 0.247 (−0.523, 1.017) | 0.530 |
| Suburban | −2.067 (−2.780, −1.353) | <0.001 | −0.462 (−1.202, 0.278) | 0.221 |
| **Average commute time to the hospital for treatment, hours** |  |  |  |  |
| <1 (ref) | — | — | — | — |
| 1.00–1.99 | 0.670 (0.028, 1.312) | 0.041 | 0.585 (0.026, 1.145) | 0.040 |
| 2.00–2.99 | −0.458 (−1.270, 0.354) | 0.269 | 0.930 (0.120, 1.740) | 0.024 |
| ≥3 | −0.167 (−1.343, 1.008) | 0.780 | 0.688 (−0.405, 1.781) | 0.218 |
| **Education** |  |  |  |  |
| Primary school or below (ref) | — | — | — | — |
| Middle school/high school/technical school | 0.137 (−1.211, 1.486) | 0.842 | −0.184 (−1.392, 1.023) | 0.765 |
| Associate/bachelor's degree | 1.720 (0.402, 3.038) | 0.011 | 0.655 (−0.635, 1.945) | 0.319 |
| Master's degree or above | 2.488 (0.970, 4.007) | 0.001 | 0.079 (−1.450, 1.609) | 0.919 |
| **Physically demanding occupations** |  |  |  |  |
| Yes (ref) | — | — | — | — |
| No | 1.828 (0.681, 2.975) | 0.002 | 0.939 (−0.232, 2.110) | 0.116 |
| Unsure | 0.594 (−0.718, 1.906) | 0.375 | 1.377 (0.162, 2.591) | 0.026 |
| **Monthly income, Chinese yuan** |  |  |  |  |
| <2000 (ref) | — | — | — | — |
| 2000–5000 | 1.158 (−0.025, 2.341) | 0.055 | 0.325 (−0.770, 1.420) | 0.561 |
| 5000–10000 | 3.592 (2.393, 4.792) | <0.001 | 1.437 (0.232, 2.641) | 0.019 |
| 10000–20000 | 3.269 (1.626, 4.912) | <0.001 | 1.133 (−0.414, 2.680) | 0.151 |
| >20000 | 2.455 (0.732, 4.177) | 0.005 | −0.812 (−2.602, 0.979) | 0.374 |
| Prefer not to disclose | 2.160 (0.899, 3.422) | <0.001 | 0.899 (−0.320, 2.117) | 0.148 |
| **Causes of pelvic pain** |  |  |  |  |
| Gynecological condition (ref) | — | — | — | — |
| Gastrointestinal condition | 0.190 (−1.476, 1.857) | 0.823 | 1.189 (−0.206, 2.584) | 0.095 |
| Urological condition | 0.220 (−1.399, 1.838) | 0.790 | 1.043 (−0.315, 2.401) | 0.132 |
| Myofascial pain | 0.833 (−1.661, 3.327) | 0.513 | 0.390 (−1.650, 2.429) | 0.708 |
| Socio-psychological condition | −0.310 (−3.000, 2.380) | 0.821 | 0.850 (−1.426, 3.126) | 0.464 |
| Neurological condition | −1.251 (−2.417, −0.085) | 0.035 | −0.859 (−1.891, 0.173) | 0.103 |
| Others | −0.596 (−1.351, 0.160) | 0.122 | −0.469 (−1.127, 0.189) | 0.163 |
| **Self-rated pain** |  |  |  |  |
| 1 (ref) | — | — | — | — |
| 2 | 1.011 (−0.289, 2.311) | 0.128 | 0.366 (−0.740, 1.471) | 0.517 |
| 3 | 0.495 (−0.848, 1.838) | 0.470 | 0.369 (−0.759, 1.497) | 0.521 |
| 4 | 1.897 (0.519, 3.275) | 0.007 | 1.180 (−0.027, 2.388) | 0.055 |
| 5 | 1.326 (0.058, 2.594) | 0.040 | 1.124 (0.028, 2.220) | 0.044 |
| 6 | 1.618 (0.390, 2.846) | 0.010 | 0.884 (−0.186, 1.955) | 0.105 |
| 7 | 1.425 (0.122, 2.728) | 0.032 | 0.015 (−1.125, 1.155) | 0.980 |
| 8 | 1.449 (−0.039, 2.937) | 0.056 | −0.235 (−1.525, 1.054) | 0.721 |

Multivariable model R² = 0.405; residual df = 556. The multivariable model included the same set of predictors retained in the primary logistic regression analysis (Table 7). Ordinary least squares linear regression was used.
